# Outcomes of bone marrow mononuclear cell transplantation combined with interventional education for autism spectrum disorder

**DOI:** 10.1002/sctm.20-0102

**Published:** 2020-09-09

**Authors:** Liem Nguyen Thanh, Hoang‐Phuong Nguyen, Minh Duy Ngo, Viet Anh Bui, Phuong T. M. Dam, Hoa Thi Phuong Bui, Doan Van Ngo, Kien Trung Tran, Tung Thi Thanh Dang, Binh Duc Duong, Phuong Anh Thi Nguyen, Nicholas Forsyth, Michael Heke

**Affiliations:** ^1^ Vinmec Research Institute of Stem Cell and Gene Technology (VRISG) Hanoi Vietnam; ^2^ Vinmec Times City International Hospital Hanoi Vietnam; ^3^ Vinmec Hightech Center Vinmec Health Care System Hanoi Vietnam; ^4^ Faculty of Medicine & Health Sciences Keele University Newcastle UK; ^5^ Department of Biology Stanford University Stanford California USA

**Keywords:** autism spectrum disorder, bone marrow mononuclear cell, stem cell transplantation, educational intervention

## Abstract

The aim of this study was to evaluate the safety and efficacy of autologous bone marrow mononuclear cell transplantation combined with educational intervention for children with autism spectrum disorder. An open‐label clinical trial was performed from July 2017 to August 2019 at Vinmec International Hospital, Hanoi, Vietnam. Thirty children who fulfilled the autism criteria of the Diagnostic and Statistical Manual of Mental Disorders, Fifth Edition, and had Childhood Autism Rating Scale (CARS) scores >37 were selected. Bone marrow was harvested by anterior iliac crest puncture under general anesthesia. The volume collected was as follows: 8 mL/kg for patients under 10 kg (80 mL + [body weight in kg − 10] × 7 mL) for patients above 10 kg. Mononuclear cells were isolated with a Ficoll gradient and then infused intrathecally. The same procedure was repeated 6 months later. After the first transplantation, all patients underwent 8 weeks of educational intervention based on the Early Start Denver Model. There were no severe adverse events associated with transplantation. The severity of autism spectrum disorder (ASD) was significantly reduced, with the median CARS score decreasing from 50 (range 40‐55.5) to 46.5 (range 33.5‐53.5) (*P* < .05). Adaptive capacity increased, with the median Vineland Adaptive Behavior Scales score rising from 53.5 to 60.5. Social communication, language, and daily skills improved markedly within 18 months after transplantation. Conversely, repetitive behaviors and hyperactivity decreased remarkably. Autologous bone marrow mononuclear cell transplantation in combination with behavioral intervention was safe and well tolerated in children with ASD (Trial registration: ClinicalTrials.gov identifier: NCT03225651).

## Graphical abstract

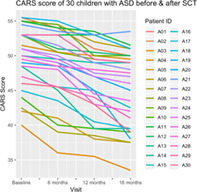

**Lessons learned**

• The combination of cell therapy and educational intervention may improve clinical manifestations such as social communication, language, and daily skills in children with ASD.

**Significance statement**

The combination of cell therapy and educational intervention may improve clinical manifestations, such as social communication, language, and daily skills, in children with autism spectrum disorder. However, additional studies with control groups should be performed in the future to obtain a more comprehensive and accurate conclusion.

## 1 INTRODUCTION

Autism spectrum disorder (ASD) is a complex spectrum of disorders characterized by two typical abnormalities: (a) deficits of social communication and interaction; (b) the presence of restricted interests as well as repetitive and stereotypic verbal and nonverbal behaviors.^1^, ^2^ Comorbidities including sleep disorders, seizures, and gastrointestinal difficulties are very common in children with ASD.^3^ The prevalence of identified ASD is increasing.^4^ In 2016, the overall ASD prevalence among children aged 4 years was 15.6 per 1000 (1/64),^5^ and the incidence was 18.5 per 1000 (1/54) in 8‐year‐old children according to Early Autism and Developmental Disabilities Monitoring Network sites.^6^ The etiology of ASD is still not well understood. However, many associated factors including genetic mutations, immune dysregulation, hypoperfusion of some parts of the brain, exposure to maternal antibodies during pregnancy, and weak functional connectivity across brain regions are suggested to contribute to the development of ASD.^7^, ^8^, ^9^, ^10^, ^11^

Multiple approaches including behavioral therapy, occupational therapy, speech therapy, and medications are required in the management of ASD to ameliorate autistic symptoms. Educational and behavioral interventions have been recognized as crucial for the management of ASD in children.^12^ The evidence indicates that young children with ASD benefit from interventions that focus on improving social interaction, communication, and challenging behaviors.^13^, ^14^ Unfortunately, many children who receive those treatments remain significantly impaired.^15^

In search of better outcomes in the management of ASD, alternative and complementary treatments are being investigated. Recent reports have suggested that stem cell transplantation result in improvements in several different neurological conditions.^16^, ^17^, ^18^ The suggested mechanisms of action of mesenchymal stem cells (MSCs) on the nervous system include neuroprotection, neurogenesis, and synaptogenesis.^19^, ^20^, ^21^

Stem cell applications were also assessed in animals using the inbred BTBR T+tf/J (BTBR) mouse strain,^22^ which has autistic‐like symptoms, to explore the potential of stem cells in the management of ASD. In BTBR mice, Segal‐Gavish et al showed that transplantation of MSCs resulted in a reduction in stereotypic behaviors, a decrease in cognitive rigidity, and an improvement in social behavior. Moreover, it has been shown that brain‐derived neurotrophic factor protein levels as well as neurogenesis increased in the hippocampus following stem cell treatment.^23^ Similarly, Perets et al demonstrated that brain‐derived neurotrophic factors secreted by transplanted MSCs were a key factor in the observed reductions in stereotypic behavior and in the improvement of cognitive flexibility in the BTBR model.^24^ Ha et al revealed that transplanted human adipose‐derived stem cells improved repetitive behaviors, social interaction, and anxiety in valproic acid‐induced ASD model mice.^25^

Based on results obtained from animal research, stem cell transplantations have been conducted for children with ASD at several centers.^26^ Two distinct approaches have been explored thus far in the application of cell therapy products for the treatment of ASD: culture expanded and nonexpanded. In an early example of non‐culture‐expanded cell therapy, Sharma et al described the use of autologous bone marrow mononuclear cell (BMMNC) transplantation infused via intrathecal route in 32 children with ASD. The procedure was reported as being safe with minor adverse events encountered, such as nausea, vomiting, and pain at the site of injection. Improvements were noticed in different aspects, including social relationships and reciprocity, speech and language patterns, and brain metabolism.^27^ A further example of concentrated, but not expanded, cell therapy described the use of autologous BMMNC transplantation via the intrathecal route in 10 children with ASD. The results revealed that the maximal treatment effect was observed within the first 12 months with, again, with no safety concerns.^28^ A combined culture‐expanded/nonexpanded cell therapy study reported on the outcomes of allogenic cord blood mononuclear cell (CBMNC) transplantation vs CBMNC combined with umbilical cord MSC transplantation for children with ASD.^29^ In this trial, four stem cell infusions were carried out via intravenous and intrathecal routes. No severe adverse events after cell transplantation were described for either group. Improved outcomes were noted in the combination group compared with the CBMNC‐alone group.

More recently, Dawson et al reported autologous stored cord blood infusion through intravenous route in 25 children with ASD, which resulted in significant improvements in behavior at 6 months after infusion; these improvements were sustained at 12 months.^30^ That study was followed by the first randomized, double‐blind, placebo‐controlled clinical trial comparing outcomes of autologous cord blood infusion vs placebo for children with ASD. As in all previous studies, autologous intravenous cord blood infusion had no serious adverse events and trended toward improvement, especially in socialization, but clinical outcomes were not significantly different between the two groups.^31^

In summary, a number of clinical trials have been performed thus far, exploring the application of cell therapy for the treatment of ASD. Although the trials have been broadly consistent in outcome reporting, disparities remain around cell sources, processing, dosage, and delivery route. The aim of this study was to investigate the safety and clinical outcomes of high‐dosage BMMNC transplantation combined with educational intervention for children with ASD.

## 2 SUBJECTS, MATERIALS, AND METHODS

### 2.1 Patients

#### 2.1.1 *Inclusion criteria*

Patients of both sexes who were aged between 3 and 7 years with a confirmed diagnosis of ASD were enrolled. ASD was diagnosed according to the diagnostic criteria for autism spectrum disorder in the Diagnostic and Statistical Manual of Mental Disorders, Fifth Edition (DSM‐5).^1^ All children at Vinmec International Hospital who had severe ASD (Childhood Autism Rating Scale [CARS] scores >37) were recruited for this study.

#### 2.1.2 *Exclusion criteria*

The exclusion criteria were epilepsy; hydrocephalus with ventricular drain; coagulation disorders; allergy to anesthetic agents; severe health conditions such as cancer or heart, lung, liver, or kidney failure; and active infections. Patients with Rett syndrome or fragile X syndrome were also excluded from this study.

### 2.2 Study design

This study was an open‐label uncontrolled clinical trial of 30 patients with ASD. The study commenced in July 2017 and was completed in August 2019. The sponsor and investigators are responsible for the publication of the trial results in accordance with the Consolidated Standards of Reporting Trials (CONSORT)^32^ guidelines.

### 2.3 Clinical assessment

Adverse events and serious adverse events were monitored and assessed throughout the entire monitoring period of the study. Complications were recorded during the process of bone marrow collections, stem cell infusions, and monitoring period of 48 hours posttransplantation, 1‐week postinjection by telephone, and then during regularly visits.

Clinical examinations were performed at baseline and then at 6, 12, and 18 months after the first transplantation by an experienced pediatric psychologist and a pediatric psychiatrist who were not members of the research team. Multiple tools were used to diagnose and determine the severity of ASD level, including DSM‐5, CARS, the Vineland Adaptive Behavior Scales Second Edition (VABS‐II), and the Clinical Global Impression (CGI). DSM‐5, which was published in May 2013, provides new diagnostic criteria for ASD. The severity of ASD is classified into three levels: level 1 (“Requiring support”), level 2 (“Requiring substantial support”), and level 3 (“Requiring very substantial support”).^33^

CARS consists of 14 domains assessing behaviors associated with ASD, with a 15th domain rating general impressions of ASD.^34^ VABS‐II is a standardized measure^35^ that yields an overall score and subscale standard scores in the four different domains: socialization, communication, daily living skills, and motor skills. CGI is a rating scale that measures symptom severity and treatment response.^36^ The severity is categorized into seven levels: (a) not present (no ASD), (b) barely evident ASD symptoms, (c) mild ASD symptoms, (d) moderate ASD symptoms, (e) moderately severe ASD symptoms, (f) severe ASD symptoms, or (g) very severe ASD symptoms. The response of each patient is also divided into seven levels: level 1, very much improved; level 2, much improved; level 3, minimally improved; level 4, no change; level 5, minimally worse; level 6, much worse; and level 7, very much worse. In addition, main indicators including social interaction, eye contact, expressive language, abnormal behaviors, sensory abnormalities, eating and sleeping difficulties, daily skills, and learning capacity before and after transplantation were collected to examine the effects of stem cell therapy in combination with behavioral intervention.

A brief video was recorded at baseline and then at 6, 12, and 18 months after the first transplantation. A questionnaire was completed by patients' caregivers 6 and 18 months after the first transplantation to obtain their assessment and satisfaction. A psychologist kept contact with patients' caregivers via telephone throughout the follow‐up period to record any unexpected events during the study.

### 2.4 Laboratory and imaging diagnostics

Routine hematologic and biochemistry examinations were performed at baseline and 6 months later. Diagnostic imaging examinations, including brain magnetic resonance imaging (MRI) and electroencephalography (EEG), were carried out in all patients at baseline to rule out epilepsy and brain malformations.

Positron emission tomography‐computed tomography (PET‐CT) was carried out before stem cell transplantation and retaken 12 months after the first transplantation to monitor changes of brain metabolism. On post hoc analysis, decreased and increased fluorodeoxyglucose (FDG) metabolism regions in autistic children were evaluated based on a normal distribution curve. If the measured value is lower than one SD from the median value of the standardized uptake value, it is considered to be hypometabolic, whereas a measured value greater than a SD from the median value is considered to be an increase in metabolism.^37^

In evaluation of PET‐CT images, dark blue brain areas were defined as severely reduced FDG metabolic rate, and light blue brain areas were assessed as moderate metabolism. Green was considered to have mild metabolic reduction. The yellow‐orange brain region was assessed to exhibit increased FDG metabolism.^38^

### 2.5 Genetic screening, data analysis, and validation

Microarray comparative genomic hybridization (CGH) and whole exome sequencing tests were performed in 28 children (two families declined genetic screening). The GenetiSure Cancer Research CGH+SNP Microarray, 2x400K Kit (Agilent Technologies, Santa Clara, California) was used for CGH testing to detect copy number variations (CNVs). This technique uses a “two‐color” process to measure DNA copy number changes in an experimental sample relative to a reference sample. An Agilent Microarray Scanner was used to scan the slides and export the data through Agilent Feature Extraction software. The first quality control filter of <0.23 derivative log‐ratio threshold was the cutoff. The ADM‐2 algorithm in Cytogenomics software was applied to all CNVs and filtered by the DGV, NSTD100, ISCA, and SFARI databases to shortlist the candidate CNVs related to ASD, and the CNVs were classified according to the American College of Medical Genetics and Genomics 2016 guidelines. The candidate CNVs were validated by real‐time polymerase chain reaction. A Rapid Capture Exome Kit (Illumina, San Diego, California) was used for library preparation. Paired‐end exome sequencing with a read length of 75 × 2 base pairs was performed on a HiSeq 4000 (Illumina). After sequencing, low‐depth reads and adapters were removed prior to downstream analysis. Burrows‐Wheeler Aligner was used for alignment using the human Genome Reference Consortium Human Build 37 (GRCh37) reference genome.^39^ The Genome Analysis Toolkit^40^ and SnpEff (an open‐source tool that annotates variants and predicts their effects on genes)^41^ were used for variant calling and annotation, respectively. Candidate variants were validated by Sanger sequencing on the ABI 3500 DX system. In silico tools, including PolyPhen‐2,^42^ Scale Invariant Feature Transform,^43^ MutationTaster,^44^ and SnpEff,^41^ were used to predict the impact of missense variants. The effect of splice site variants was predicted by Human Splicing Finder.^45^

### 2.6 Bone marrow aspiration

For each transplantation, bone marrow was harvested through anterior iliac crest puncture under general anesthesia in the operating theater. The volume collected depended on the patients' body weight as follows: 8 mL/kg for patients under 10 kg (80 mL + [body weight in kg − 10] × 7 mL) for patients above 10 kg, based on our experience from a previous study.^17^

### 2.7 BMMNC isolation, characterization, and preparation for transplantation

BMMNCs were isolated by gradient centrifugation using Ficoll‐Paque (GE Healthcare, Waukesha, Wisconsin) in our ISO 14644 standard clean room at Vinmec Research Institute of Stem Cell and Gene Technology. The cell suspension was washed with 1× phosphate‐buffered saline solution and resuspended in autologous plasma up to a total of 10 mL for injection. The sterility of the product was confirmed by microbiological evaluation using the BacT/Alert3D microbial detection system (bioMérieux, Durham, North Carolina). The total blood components before and after Ficoll‐Paque separation were evaluated with a Beckman Coulter LH780 hematocytometer. The hematopoietic stem cell CD34^+^ (hHSC CD34^+^) count was assessed using Stem‐Kit Reagent (Beckman Coulter, Brea, California) on a Navios flow cytometer (Beckman Coulter). Before injection, cell products were examined for endotoxin levels using the Endosafe‐PTS kit (Charles River Laboratories, Cambridge, Massachusetts) and Mycoplasma using the MycoAlert Mycoplasma Detection Kit (Lonza, Basel, Switzerland).

### 2.8 BMMNC transplantation

Each patient underwent two BMMNC transplantations with an interval of 6 months. The average mononuclear cell and CD34^+^ cell counts per kg body weight were 42.3 × 10^6^/kg and 2.6 × 10^6^ for the first transplantation and 40.9 × 10^6^/kg and 2.1 × 10^6^ for the second transplantation, respectively. The average cell viabilities before the first and second transplantations were 97.6% and 98.7%, respectively. Each dose of cells was mixed with physiological saline to a volume of 5 mL for administration. Cells were then intrathecally infused into the space between the fourth and fifth lumbar vertebrae using an 18‐gauge needle. This procedure was conducted in the recovery room by an experienced anesthesiologist and lasted 30 minutes.

### 2.9 Post‐BMMNC transplantation therapy

All patients underwent 8 weeks of educational intervention, 2 hours per day, 5 days per week, at the day clinic of Vinmec International Hospital. The educational intervention was developed based on the Early Start Denver Model manual.^15^, ^46^

### 2.10 Ethics statement

The study protocol was reviewed and approved by the Ethics Committee of Vietnam Ministry of Health on 20 June 2017, with number 72/CN‐BDGDD. The study was registered on ClinicalTrials.gov on 21 July 2017, with identity number NCT03225651.

The study protocol was reviewed and approved by the Institutional Review Board of Vinmec International Hospital on 31 March 2017. The reference number for the ethics committee is 310317/2017/QD‐VINMEC. The committee evaluated the ethical aspects of the study in accordance with The World Medical Association's Declaration of Helsinki. The study was explained in detail to the parents of the participants. Written informed consent was obtained from the parents well before patient enrollment in all cases. Written informed consent was obtained from the subjects' parents well before patient enrollment in all cases. This consent included their agreement to the publication of indirect patient identifiers, such as age and gender.

### 2.11 Statistical analysis

Each individual is a unit of analysis. The Wilcoxon signed‐rank test was used to compare the total CARS scores and VABS scores at 6, 12, and 18 months with those at baseline. Mixed‐effects analysis was used to evaluate the changes in CARS scores. A value of *P* < .05 was considered statistically significant. All statistical analyses were performed using R software version 3.4.4.

## 3 RESULTS

### 3.1 Patient characteristics

Thirty patients, including 25 boys and 5 girls with a mean age of 5.6 ± 0.9 years (range 3.0‐7.4 years) and a mean weight of 19.9 ± 3.6 kg, were enrolled in this study. All patients underwent educational intervention before the first transplantation with an average duration of 34 ± 17.5 months. The age when those children received such educational intervention was 26.4 ± 5.8 months at different centers.

At baseline, all patients were categorized at a severe level with CARS scores ranging from 40 to 55.5. According to the DSM‐5 classification, 28 patients were classified as level 3, and 2 patients were classified as level 2. CARS scores and VABS scores at baseline are shown in Table 1.

**TABLE 1:** Median Childhood Autism Rating Scale (CARS) and Vineland Adaptive Behavior Scale scores at baseline and after transplantation (N = 30)

Category	Baseline, median (range)	After 6 months, median (range)	After 12 months, median (range)	After 18 months, median (range)
CARS score	50 (40‐55.5)	49.5 (36‐55)*	47.3 (35.5‐53.5)*	46.5 (33.5‐53.5)*
Vineland standard score	53.5 (40‐65)	55.5 (42‐66)*	58.5 (43‐71)*	60.5 (44‐76)*
Communication	47 (38‐67)	47 (36‐74)	51 (36‐71)*	56 (36‐79)*
Daily living skills	58 (38‐75)	58 (36‐79)	65.5 (40‐85)*	67.6 (40‐89)*
Socialization	55 (46‐65)	53 (44‐68)	59 (46‐68)*	59 (50‐74)*
Motor skills	64 (38‐78)	64 (49‐81)	64 (52‐81)	67 (51‐89)*

* *P* < .05 (Wilcoxon signed‐rank test compared with baseline).

Adaptive behavior skills were low in all patients, with VABS scores ranging from 41 to 65. Before BMMNC transplantation, only 37% of the children could have social contact, and 23% made eye contact; meanwhile, the rates of no eye contact and not showing affection toward parents were 77% and 87%, respectively. Expressive language occurred in 47% of children (Table 2).

**TABLE 2:** Social interaction, eye contact, and expressive language before and after transplantation (N = 30)

Domain	Before transplantation, N (%)	After 6 months, N (%)	After 12 months, N (%)	After 18 months, N (%)
Social interaction				
No social interaction	19 (63)	12 (40)	7 (23)	1 (3)
Capable of social interaction	11 (37)	18 (60)	23 (77)	29 (97)
Eye contact				
No eye contact	23 (77)	17 (57)	4 (13)	2 (7)
Occasional or normal eye contact	7 (23)	13 (43)	26 (87)	28 (93)
Expression of feelings to parents				
Showing affection	26 (87)	26 (87)	29 (97)	29 (97)
Not showing affection	4 (13)	4 (13)	1 (3)	1 (3)
Expressive language				
No language	16 (53)	10 (33)	4 (13)	2 (7)
Capable of expressive language	14 (47)	20 (67)	26 (87)	28 (93)

Abnormal behaviors were observed in a high percentage of children. Regarding stereotypic behaviors, 93% of the children displayed repetitive behavior and 83% restricted interest. Hyperactivity was noted in 28 patients (93%) (Table 3). Sensory abnormalities occurred in 29 children (97%), picky eating in 21 children (70%), and sleeping difficulties in 16 children (53%) (Table 4).

**TABLE 3:** Abnormal behaviors before and after transplantation (N = 30)

Domain	Before transplantation, N (%)	After 6 months, N (%)	After 12 months, N (%)	After 18 months, N (%)
Stereotypic/repetitive behaviors				
Have repetitive behavior	28 (93)	26 (87)	26 (87)	26 (87)
No repetitive behavior	2 (7)	4 (13)	4 (13.3)	4 (13)
Restricted interests				
Have restricted interests	25 (83)	25 (83)	23 (77)	22 (73)
No restricted interests	5 (17)	5 (17)	7 (23)	8 (27)
Hyperactivity				
Have hyperactivity	28 (93)	27 (90)	21 (70)	13 (43)
No hyperactivity	2 (7)	3 (10)	9 (30)	17 (57)
Self‐injurious behavior				
Have self‐injurious behavior	6 (20)	6 (20)	5 (17)	2 (7)
No self‐injurious behavior	24 (80)	24 (80)	25 (83)	28 (93)

**TABLE 4:** Sensory abnormalities, eating difficulties and sleep problems before and after transplantation (N = 30)

Domain	Before transplantation, N (%)	After 6 months, N (%)	After 12 months, N (%)	After 18 months, N (%)
Sensory impairments				
Have sensory abnormalities	29 (97)	28 (93)	24 (80)	22 (73)
No sensory abnormalities	1 (3)	2 (7)	6 (20)	8 (27)
Picky eating behaviors				
Have picky eating behavior	21 (70)	20 (67)	18 (60)	14 (47)
No picky eating behavior	9 (30)	10 (33)	12 (40)	16 (53)
Sleep problems				
Have sleep problems	16 (53)	15 (50)	14 (47)	13 (43)
No sleep problems	14 (47)	15 (50)	16 (53)	17 (57)

### 3.2 Genetic testing

We detected and validated a de novo CNV in the *SHANK3* gene from a female proband (proband A27) and 23 different variants in 22 genes from eight probands (Table 5). Among them, 15 variants were recorded in dbSNP, most of which have not been reported to have clinical significance. TRIOS analysis revealed the inheritance mode of the variants where two probands (probands A11 and A13) carried autosomal recessive variants, three probands carried X‐linked variants (A17, A22, and A29), and the rest carried de novo variants. In terms of variant type, although most of them were missense, we observed three loss‐of‐function variants (two frameshift and one deletion) in three male probands (proband A6, A13, and A29). Our in silico analyses showed that almost all variants had a damaging impact on the gene's function. We found that 41% of the total detected genes harboring variants were recorded in the ASD database, including *ANK2*, *CASK*, *CHD8*, *GALNT14*, *GIGYF2*, *GRIN2A*, *MUC4*, *NOS1*, and *XIRP1*. *SHANK3*, *CHD8*, *ANK2*, and *GIGYF2* belong to the genes with the strongest evidence of relevance to ASD.

**TABLE 5:** Detected genetic variations

Proband (gender)	Chromosome	Gene	Variant		Impact			Inheritance	CARS score	
			Variant ID	Base change	PolyPhen‐2 (score)	Mutation taster (score)	SIFT (score)		Before transplant	After transplant
A3 (F)	12	*IGF1*		c.251G>A	D(0.998)	D(1)	D(0.001)	De novo	40	33.5
A6 (M)	3	*MUC4*		c.332_333insACGCA				De novo	55	51
A11 (F)	7	*PLOD3*	rs138610113	c.1315G>A	P(0.625)	D(0.999)	D(0.001)	AR	55	51.5
A13 (M)	2	*GIGYF2*	rs371622656	c.3695_3696insGC				AR	53	50
A17 (M)	X	*CASK*		c.2482G>A	P(0.682)	D(1)	D(0.002)	X‐linked	45	39.5
	14	*CHD8*		c.3575T>C	D(0.948)	D(1)	D(0)	De novo		
A22 (M)	X	*GPC3*	rs148021273	c.359G>A	D(0.985)	D(0.998)	D(0.005)	X‐linked	53	48
A27 (F)	22	*SHANK3*	Copy number variation, whole‐gene deletion					De novo	49.5	47.5
	14	*TSHR*	rs540799629	c.1838A>G	D(0.989)	D(1)	D(0.015)	De novo		
	5	*DNAH5*	rs561666802	c.7452C>A	B(0.034)	N(0.927)	D(0.017)	De novo		
	3	*XIRP1*	rs373538005	c.1135C>T	D(0.916)	D(0.994)	D(0.001)	De novo		
	1	*AGL*	rs146257941	c.4075C>T	D(0.995)	D(1)	D(0)	De novo		
	16	*ERCC4*		c.2009G>T	D(0.948)	D(1)	D(0.002)	De novo		
	2	*GALNT14*	rs199694822	c.445C>T	P(0.571)	N(0.916)	D(0.002)	De novo		
	17	*XYLT2*	rs199498175	c.289C>T	D(0.998)	D(0.951)	D(0.001)	De novo		
	12	*NOS1*	rs375371439	c.3374C>T	D(1)	D(1)	D(0.031)	De novo		
	16	*GRIN2A*	rs201072838	c.2566C>G	B(0.405)	D(0.819)	D(0.007)	De novo		
	18	*PIGN*	rs576797024	c.896A>G	P(0.893)	D(1)	T(0.486)	De novo		
	18	*PIGN*	rs373378832	c.49G>A	B(0.004)	N(0.758)	T(0.248)	De novo		
	4	*ANK2*	rs3733616	c.7267G>A	B(0.033)	D(0.981)	D(0.001)	De novo		
	22	*TMPRSS6*		c.1016A>C	D(0.998)	D(1)	D(0.008)	De novo		
	4	*PIGG*	rs141963092	c.2891T>C	P(0.548)	D(0.940)	D(0.002)	De novo		
A29 (M)	X	*SYP*		c.251C>G	P(0.816)	D(1)	D(0.002)	X‐linked		
	X	*LAS1L*		c.1797_1805delTGATGAAGA				X‐linked	53	49

Abbreviations: AR, autosomal recessive; CARS, Childhood Autism Rating Scale; F, female; M, male; SIFT, Scale Invariant Feature Transform.

### 3.3 Brain MRI and EEG

No abnormalities on MRI or EEG were observed in any of the patients.

### 3.4 FDG changes after BMMNC transplantation

At baseline, manifestation of hypometabolism was found at seven major brain regions: hippocampus, anterior cingulate gyrus, posterior cingulate gyrus, parietal lobe, frontal lobe, temporal lobe, and central sulcus. After the stem cell transplant, 29 children underwent a second PET‐CT scan (the parent refused the second PET‐CT in one patient). Improvement in metabolism was observed in some brain regions where severe hypometabolism was noticed before BMMNC transplantation such as parietal lobe, frontal lobe, and anterior cingulate gyrus. However, these changes were not statistically significant (Table 6).

**TABLE 6:** Brain metabolism of autistic children before and after stem cell transplantation as assessed by positron emission tomography‐computed tomography examination (N = 29)

Regions	Severe hypometabolism (%)		Moderate hypometabolism (%)		Light hypometabolism (%)		No change (%)	
	Before (N = 29)	After (N = 29)	Before (N = 29)	After (N = 29)	Before (N = 29)	After (N = 29)	Before (N = 29)	After (N = 29)
Hippocampus	3.4	0	41.4	27.6	17.2	66	38	6.4
Anterior cingulate gyrus	31	13.8	48.3	51.7	10.3	10.3	10.3	24.2
Posterior cingulate gyrus	6.9	3.4	10.3	3.4	6.7	0	76.1	93.2
Parietal lobe	24.1	0	31	13.8	10.3	31	34.6	55.2
Frontal lobe	24.1	3.4	24.1	0	3.4	31	48.4	65.6
Temporal lobe	6.9	0	37.9	10.3	10.3	20.7	44.9	69
Central sulcus	0	0	3.4	10.3	3.4	27.6	93.2	62.1

### 3.5 Adverse events

None of the patients had any severe adverse events during bone marrow aspiration, stem cell infusion, or following transplantation. The procedure was reported as being safe with minor adverse events encountered. There were no procedure‐related major adverse events. Among 96 adverse events that occurred during study period, 46 (48%) mild and moderate adverse events were recorded that may or may not be related to BMMNC transplantation with symptoms including pain, vomiting, and mild fever. All those adverse events were easily managed through appropriate medication (Table 7).

**TABLE 7:** Summary of the number of adverse events (AEs) and serious adverse events (SAEs) occurring during the study

AE/SAE	N (%)	Classification of AE/SAE	Note
Serious adverse events	0	SAE	
AE was not associated with the intervention	26 (27.1)	AE	Nonallergic rhinitis, tonsillitis, cold urticaria, leg pain, poor appetite, abrasions
AE was less associated with the intervention	27 (28.1)	AE	Skin rash, pale skin, pale mucous membranes, fussing, lack of sleep, fatigue
AE may be related to the intervention	17 (17.7)	AE	Mild fever, nausea, vomiting
AE related to the intervention	26 (27.1)	AE	Pain, broken vein, peripheral vein masonry, slipping needle out of the vein during transplantation
Total	96		

### 3.6 Clinical outcomes

**FIGURE 1:**
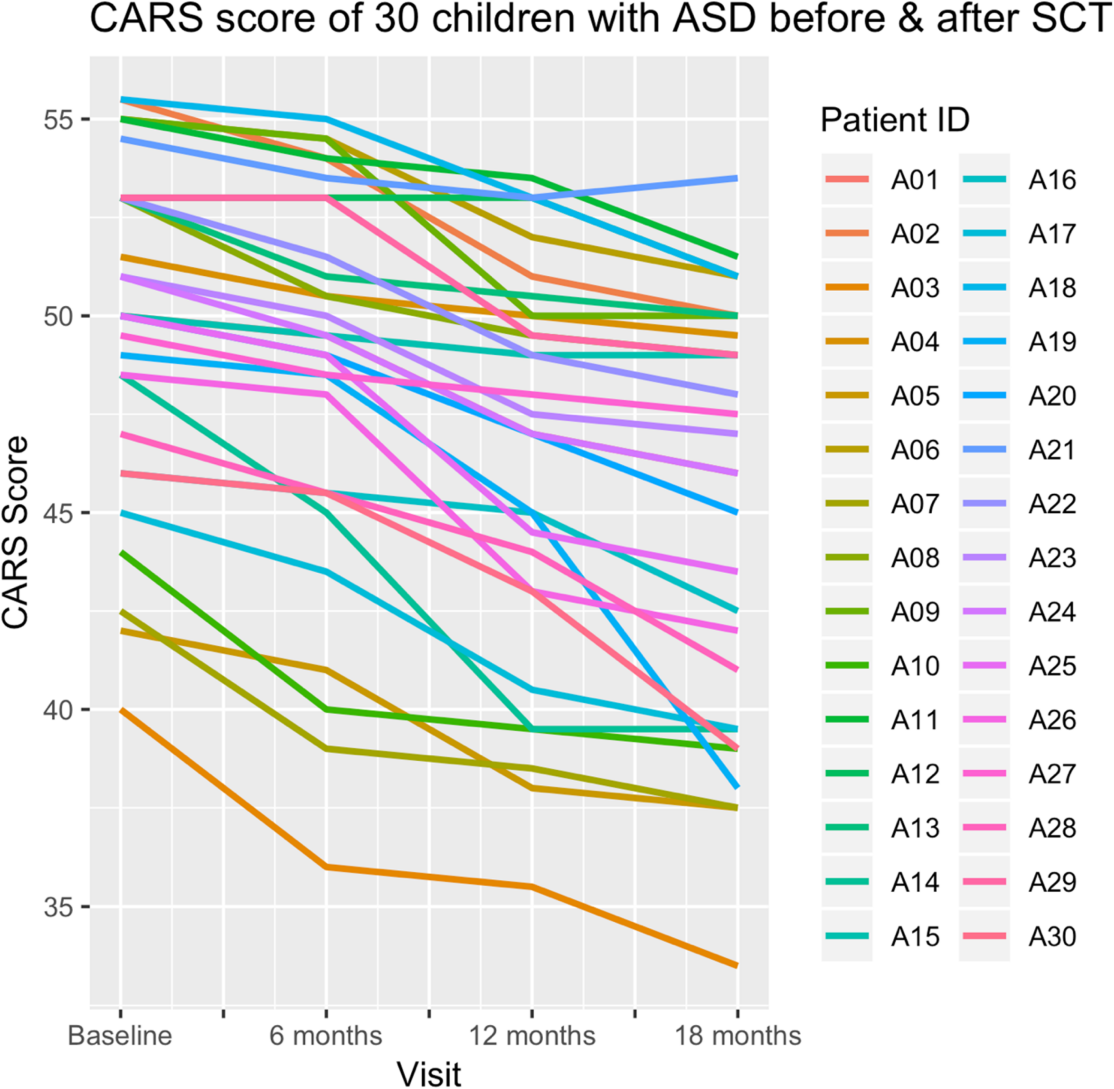
Childhood autism rating scale (CARS) scores for 30 children with autism spectrum disorder (ASD) before and after stem cell transplantations (SCTs)

After BMMNC transplantation, the severity of ASD decreased remarkably. The median CARS scores decreased from 50 (range 40‐55.5) points at baseline to 46.5 (range 33.5‐53.5) after 18 months. Patient‐specific CARS score analysis for each patient is presented in Figure 1. The result of the mixed‐effects analysis suggests that each visit was associated with a decrease of 1.6 in the CARS score and that this change was statistically significant (Table 8).

**TABLE 8:** The results from the mixed‐effects analysis of Childhood Autism Rating Scale scores

Fixed effects	Estimate ± SE	*t* value
(Intercept)	51.60000 ± 0.91245	56.55
Visit	−1.62167 ± 0.09957	−16.29
**Correlation of fixed effects**		
	(Intr)	
Visit	−0.273	

According to the DSM‐5 classification, the number of patients at level 3 was reduced from 28 to 18. There were no patients at level 1 before transplantation, but after transplantation, five children were recategorized at this level.

After transplantation, improvements were observed in various aspects. Social interaction and eye contact increased remarkably from 37% before the first transplantation to 97% and from 23% to 93%, respectively, after 18 months. Expressive language increased from 47% before transplantation to 67% after 6 months, 87% after 12 months, and 93% after 18 months (Table 2). Abnormal behaviors also decreased after transplantation. The children with repetitive behavior decreased from 93% to 87% (Table 3). Sensory abnormalities and sleeping difficulties progressively improved after transplantation, from 97% to 73% and from 53% to 43%, respectively (Table 4). Improvements of adaptive behavior scales after transplantation are noted in VABS scores, including communication, daily living skills, and socialization. Specifically, the Vineland standardized scores increased significantly from 52.4 ± 7 to 60.4 ± 7.4. This increase in adaptive ability was found in all four different domains: from 49 ± 8.3 to 56.1 ± 9.8 in communication, from 55.7 ± 10.1 to 67.1 ± 12 in daily living skills, from 54.5 ± 5.4 to 58.9 ± 5.5 in socialization, and from 60.1 ± 14.7 to 67.8 ± 8.6 in motor skills (Table 1).

According to teachers' evaluations, 28 children exhibited improvements compared with baseline, including 5 patients who improved very much, 17 who improved much, and 6 who improved minimally. Two children exhibited very minimal changes when compared with the baseline assessment. In addition, parents' satisfaction with the intervention increased from 93% at 6 months to 97% at 18 months of BMMNC treatment.

## 4 DISCUSSION

In this study, bone marrow was collected from anterior iliac crests and not from posterior crests as in other reports.^17^, ^27^ This change facilitated anesthesia and reduced risks related to the prone position of patients. The dosage of transplanted mononuclear cells and CD34^+^ cells in our study was higher than those in studies reported by Sharma, Dawson, and Chez.^27^, ^30^, ^31^ Differences related to the cell transplants compared with other studies are presented in Table 9. Granulocyte‐colony stimulating factor (G‐CSF) was not used in this study to mobilize stem cells from bone marrow, with high dosage values nevertheless isolated. This implies that G‐CSF may not be necessary for the collection of BMMNCs in children. Our results demonstrated that high‐dosage transplantation of autologous bone marrow stem cells was well tolerated. There were no observed severe adverse events in any patients during or following transplantation. The incidence of minor adverse events was low and easily managed through medication or spontaneously resolved themselves.

**TABLE 9:** Number of cells by study

Author	Cell type	Number of cells	Number of CD34^+^ cells	Route
Chez et al^31^	Autologous umbilical cord blood mononuclear cell	16.16 × 10^7^ cells/kg	0.29 × 10^5^ cells/kg	Peripheral i.v.
Dawson et al^30^	Autologous umbilical cord blood mononuclear cell	2.6 × 10^7^ cells/kg	0.3 × 10^5^ cells/kg	Intravenous
Sharma et al^27^	Autologous BMMNC	8.9 × 10^7^ cells	—	Intrathecal
Liem et al^47^	Autologous BMMNC	First dose: 42.3 × 10^6^ MNCs/kg Second dose: 40.9 × 10^6^ MNCs/kg	First dose: 2.6 × 10^6^ cells/kg Second dose: 2.1 × 10^6^ cells/kg	Intrathecal

Abbreviations: BMMNC, bone marrow mononuclear cell; MNC, mononuclear cell.

Although all participants still belonged to severe level at the baseline after receiving behavioral intervention with a mean duration of 3.5 years, this study showed improvements in various aspects after BMMNC transplantation combined with educational intervention. Positive changes in social communication, eye contact, language, behaviors, and daily skills were observed after BMMNC transplantation. In addition, learning ability also remarkably improved after transplantation. The number of children who could go to school without support increased after transplantation (Table 10).

**TABLE 10:** Daily skills and learning capacity before and after transplantation (N = 30)

Domain	Before transplantation, N (%)	After 6 months, N (%)	After 12 months, N (%)	After 18 months, N (%)
Self‐feeding				
Unable to self‐feed	13 (43)	4 (14)	1 (3)	1 (3)
Need support to self‐feed	13 (43)	16 (53)	11 (37)	10 (33)
Able to self‐feed	4 (14)	10 (33)	18 (60)	19 (64)
Toileting skills				
Unable to go to toilet	17 (56)	12 (40)	7 (23)	6 (20)
Need support to go to toilet	11 (37)	14 (47)	11 (37)	10 (33)
Able to go to toilet	2 (7)	4 (13)	12 (40)	14 (47)
Learning ability				
Unable to integrate into school	16 (53)	15 (50)	11 (37)	8 (27)
Need support to integrate into school (with help from teachers)	14 (47)	15 (50)	18 (60)	19 (63)
Go to school normally	0	0	1 (3)	3 (10)

Hyperactivity of children with ASD is a disorder that severely impairs quality of life for the whole family. In our study, the rate of children with hyperactive disorder decreased by 50% at 18 months after stem cell transplantation.

Positive changes were found in evaluation measures, including severity and adaptive ability. The number of patients at level 3 (requiring very substantial support) according to DSM‐5 decreased from 28 to 18 at 18 months after transplantation.

We noticed that the improvements appeared to be influenced by the CARS scores at baseline. Patients with a CARS score ≤49 at baseline showed better improvement than those who had CARS scores >49 points. This would imply that patients with lesser severity had better outcomes after transplantation.

Genetic abnormalities have been reported in many ASD studies. In our series, genetic variations were found in eight patients (26%). Among the detected genes, *CHD8*, *ANK2*, and *SHANK3* have been well identified as ASD risk genes.^48^, ^49^ Meanwhile, some genes have been found to associate with neurodevelopmental disorders (*CASK* and *CHD8*)^50^ or to be involved in synapse transmission, such as *IGF1* (proband A3),^51^ *PIGG* (proband A27),^52^ *SYP*,^53^ and *LAS1 L*.^54^ Among those eight children, clinical improvements were observed in seven patients at different levels after BMMNC transplantation, whereas one patient (proband A27), who carried multiple genetic abnormalities, exhibited the least progress.

Stem cell transplantations display effects in some studies linked to ASD; however, there are still ongoing controversial discussions concerning the most appropriate stem cell source, preparation, stem cell dosage, delivery routes, and study follow‐up schedules after transplantation. Different sources of stem cells have been explored as potential cell therapies for ASD, including umbilical CBMNCs, umbilical cord tissue MSCs, and BMMNCs.^27^, ^28^, ^29^, ^30^, ^31^

The safety and efficacy of autologous BMMNC transplantation has been shown for many neurologic conditions.^27^, ^28^, ^47^, ^55^, ^56^ We propose that autologous BMMNCs are a suitable cell source for application in the management of ASD.

Two different pathways for administration have been applied in the delivery of cell therapy for ASD: intravenous and intrathecal. Although both delivery routes are safe, there are concerns regarding cells delivered through the intravenous route, as transplantations in animal models have shown that the transplanted cells have difficulties passing through organs such as the spleen, kidney, and intestine.^57^ The intrathecal route does not present this concern. The number of BMMNCs transplanted also varies between studies, and the number we have used was higher than those from other groups.^27^, ^28^, ^29^, ^30^, ^31^ There have been suggestions of correlation between transplanted stem cell dosage and the extent of subsequent clinical improvement.^58^ Although we did not perform an escalating dosage study, we noted that a high dosage of stem cells may be used to obtain satisfactory outcomes in children with ASD. Furthermore, we performed two transplantations instead of one transplantation as described elsewhere.^27^, ^30^, ^31^ Multiple transplantations have resulted in positive outcomes in both ASD^29^ and spinal cord injury.^59^ Moreover, the benefits of repeated transplantations vs single transplantation have been identified in animal myocardium infarction models.^60^

In our study, all children received 18 months of follow‐up after the first transplantation. This follow‐up duration exceeds those in other recent reports.^27^, ^29^, ^30^, ^31^ We noticed that the longer the follow‐up duration was, the lower the severity of ASD (CARS score reduction) and the better the children's adaptive functioning (VABS score increase). Meanwhile, there was no case in which the results were not improved or even worse compared with baseline, implying that the treatments have a sustainable long‐term effect. It is likely that further extended follow‐up times will be required to fully assess the responses of children with ASD over time after stem cell transplantation. Herein, we have demonstrated the safety and feasibility of BMMNC transplantation for the treatment of ASD. The lack of a control group who received either stem cell transplantation or educational intervention only is a limitation of this study. However, our study results provide initial evidence to justify conducting a randomized clinical trial with control groups in the future.

## 5 CONCLUSION

We conclude that autologous BMMNC transplantation is safe. The combination of cell therapy and educational intervention may improve clinical manifestations such as social communication, language, and daily skills in children with ASD. However, additional studies with control groups should be performed in the future to obtain a more comprehensive and accurate conclusion.

## CONFLICT OF INTEREST

M.H. declared an advisory role for Regenerative Medicine at Vinmec International Hospital. The other authors declared no potential conflicts of interest.

## AUTHOR CONTRIBUTIONS

L.N.T.: conception and design, collection and/or assembly of data, data analysis and interpretation, manuscript writing, final approval of manuscript; H.‐P.N., M.N.D., A.B.V., P.T.M.D.: conception and design, collection and/or assembly of data, data analysis, manuscript writing, final approval of manuscript; H.B.T.P., D.N.V., K.T.T., T.D.T.T., B.D.D., A.N.T.P., N.F., M.H.: collection and/or assembly of data, data analysis and interpretation, manuscript writing, final approval of manuscript.

## Data Availability

All data generated or analyzed during this study are included in this published article and its supplementary information files.
